# New Diagnostic Modality Combining Mass Spectrometry and Machine Learning for the Discrimination of Malignant Intraductal Papillary Mucinous Neoplasms

**DOI:** 10.1245/s10434-022-13012-y

**Published:** 2023-01-08

**Authors:** Sho Kiritani, Tomohiko Iwano, Kentaro Yoshimura, Ryo Saito, Takashi Nakayama, Daisuke Yamamoto, Hiroyuki Hakoda, Genki Watanabe, Nobuhisa Akamatsu, Junichi Arita, Junichi Kaneko, Sén Takeda, Daisuke Ichikawa, Kiyoshi Hasegawa

**Affiliations:** 1 Hepato-Biliary-Pancreatic Surgery Division, Department of Surgery, Graduate School of Medicine, The University of Tokyo, Tokyo, Japan; 2 Department of Anatomy and Cell Biology, Faculty of Medicine, The University of Yamanashi, Yamanashi, Japan; 3 Division of Molecular Biology, Center for Medical Education and Sciences, Interdisciplinary Graduate School of Medicine, University of Yamanashi, Yamanashi, Japan; 4 Department of Digestive Surgery and Breast and Endocrine Surgery, The University of Yamanashi, Yamanashi, Japan; 5 Department of Anatomy, Teikyo University School of Medicine, Tokyo, Japan

## Abstract

**Background:**

An intraductal papillary mucinous neoplasm (IPMN) is a pancreatic tumor with malignant potential. Although we anticipate a sensitive method to diagnose the malignant conversion of IPMN, an effective strategy has not yet been established. The combination of probe electrospray ionization-mass spectrometry (PESI-MS) and machine learning provides a promising solution for this purpose.

**Methods:**

We prospectively analyzed 42 serum samples obtained from IPMN patients who underwent pancreatic resection between 2020 and 2021. Based on the postoperative pathological diagnosis, patients were classified into two groups: IPMN-low grade dysplasia (n = 17) and advanced-IPMN (n = 25). Serum samples were analyzed by PESI-MS, and the obtained mass spectral data were converted into continuous variables. These variables were used to discriminate advanced-IPMN from IPMN-low grade dysplasia by partial least square regression or support vector machine analysis. The areas under receiver operating characteristics curves were obtained to visualize the difference between the two groups.

**Results:**

Partial least square regression successfully discriminated the two disease classes. From another standpoint, we selected 130 parameters from the entire dataset by PESI-MS, which were fed into the support vector machine. The diagnostic accuracy was 88.1%, and the area under the receiver operating characteristics curve was 0.924 by this method. Approximately 10 min were required to perform each method.

**Conclusion:**

PESI-MS combined with machine learning is an easy-to-use tool with the advantage of rapid on-site analysis. Here, we show the great potential of our system to diagnose the malignant conversion of IPMN, which would be a promising diagnostic tool in clinical settings.

**Supplementary Information:**

The online version contains supplementary material available at 10.1245/s10434-022-13012-y.

An intraductal papillary mucinous neoplasm (IPMN) of the pancreas is characterized by the production of mucin with a dilated pancreatic duct.^1-3^ This category of pancreatic tumor was first conceptualized in the 2^nd^ section of the World Health Organization classification in 1996, which made clinicians aware of IPMN as a differential diagnosis. Since then, the number of IPMN cases has increased because of the enhanced accuracy of imaging technology and recognition of the disease.^4,5^

An intraductal papillary mucinous neoplasm (IPMN) of the pancreas is characterized by the production of mucin with a dilated pancreatic duct.^1-3^ This category of pancreatic tumor was first conceptualized in the 2^nd^ section of the World Health Organization classification in 1996, which made clinicians aware of IPMN as a differential diagnosis. Since then, the number of IPMN cases has increased because of the enhanced accuracy of imaging technology and recognition of the disease.^4,5^

IPMN is pathologically divided into three classes: low-grade dysplasia (IPMN-LGD), high-grade dysplasia (IPMN-HGD), and invasive carcinoma (IPMN-IC).^6-8^ IPMN-HGD is considered potentially malignant and is therefore treated as an indication for surgery, similar to invasive carcinoma.^9^ The international Fukuoka criteria coined words, such as “high-risk stigmata (HRS)” and “worrisome feature (WF),” to discern IPMN-HGD from IPMN-LGD.^10^ However, there are no definitive methods to predict their properties more accurately, and the latest method achieves a diagnostic accuracy of no more than 63–76%.^11-13^

Recently, liquid biopsy has received great attention worldwide because of its relatively higher sensitivity in detecting the early phase of malignant conversion. Serological analysis by conventional mass spectrometry still contributes to cancer screening, but because of its inherent drawbacks in procedures, such as low throughput analysis, complicated sample preparation, and chromatographic separation, these techniques play a supporting role. Probe electrospray ionization-mass spectrometry (PESI-MS) is a derivative of ESI that uses a unique needle ion emitter without a troublesome capillary.^14^ We took advantage of PESI-MS to develop a medical device that is useful for cancer diagnosis.^14,15^ Based on this perspective, we applied this to various malignancies, such as head and neck, stomach, liver, and breast cancer.^16-19^

The aim of this study was to verify the diagnostic power of this system to accurately predict IPMN subtypes by detecting tumor-specific serological changes.

## Materials and Methods

### Patient Selection and Data Collection

This study was conducted in accordance with the ethical standards of the Declaration of Helsinki, and the protocol was approved by the ethics committees of the University of Tokyo and the University of Yamanashi [approval numbers: 2019370NI-(2) and 2086]. Each patient provided written informed consent before participating in this study, and all clinicopathological data were anonymized.

We examined 42 IPMN patients who did not have histologically proven cancer before resection, and who underwent pancreatic resection between February 2020 and November 2021 at the University of Tokyo Hospital and the University of Yamanashi Hospital. Preoperative tumor evaluation was basically performed by endoscopic ultrasonography. Endoscopic retrograde cholangiopancreatography was added as needed. We regularly assessed the diameter of the cyst and the presence of an enhanced mural nodule by endoscopic ultrasonography, but we did not always perform aspiration for the cyst. If endoscopic retrograde cholangiopancreatography was performed, pancreatic juice cytology was simultaneously performed. Surgical indications were as follows: main-duct type IPMN, combined-duct type IPMN, branched-duct type IPMN with high-risk stigmata, and malignant suspicious branched-duct type IPMN with worrisome features.^10^ Clinicopathological data from patients were acquired from a prospectively maintained database. Pathological findings were evaluated based on IPMN international guidelines.^10^ We defined IPMN-HGD and IPMN-IC as advanced IPMN (Ad-IPMN) and compared IPMN-LGD and Ad-IPMN.

### Sample Collection and Preparation

The blood draw was performed just prior to the surgery. Venous blood samples from 42 IPMN patients were preoperatively infused into thrombin-containing tubes by experienced phlebotomists. After the tube remained upright for 15–60 min at room temperature, it was centrifuged at 3000 rpm for 5 min. Then, the supernatant was collected and frozen at −80 °C until PESI-MS analysis.

Ten microliters of dissolved serum samples were added to 390 µl 50% ethanol and stirred using a vortex mixer (Scientific Industries, Inc.) at 10 °C for 5 min. After being placed on ice for 5 min, the sample was centrifuged at 15,000 rpm for 10 min at 4 °C. Nine microliters of supernatant were added to a sample plate (Shimadzu, Corp.)

### PESI-MS Analysis and Data Processing

**Fig. 1:**
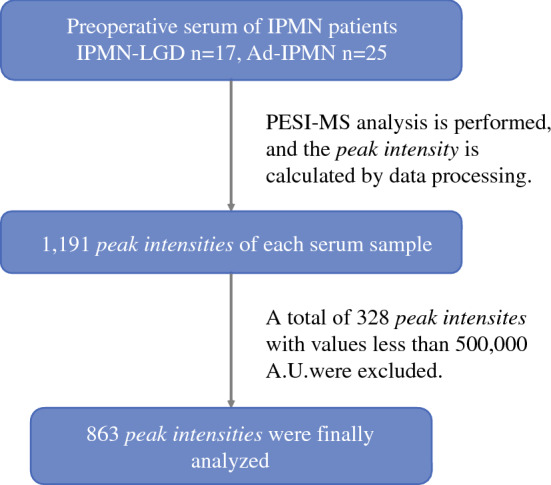
The patient flowchart for this study. A total of 1191 peak intensities were obtained from each patient. Among them, 328 peak intensities with values influenced by electronic noise were excluded. The remaining 863 peak intensities were analyzed. *IPMN*, intraductal papillary mucinous neoplasm; *LGD*, low-grade dysplasia; *Ad*, Advanced

A Direct Probe Ionization-Mass Spectrometer-8060 (DPiMS, Shimadzu, Corp.) was used for the analysis. The detailed method of operation of this instrument has been described in previous reports.^18-21^ The ion intensity was obtained in positive ion mode using this mass spectrometer and analyzed by LabSolutions software (Shimadzu, Corp.). The mass spectrum was visualized by *m/z* (the mass to charge ratio) in the *x*-axis and ion intensity (A.U.) in the *y*-axis. To obtain the peak value defined as the “peak intensity,” the ion-intensity value was added to the maximum ion intensity within one *m/z* and the values before and after it. The corresponding *m/z* was rounded down to an integer bin. An example of data processing is shown in Supplementary Fig. 1. Bins with a peak intensity less than 500,000 A.U. were excluded from the analysis. This value almost corresponded to the quartile of IPMN data and corresponded to the maximum data of the solvent-only sample (Supplementary Fig. 2). With the above processing, 328 out of 1191 peak intensities were excluded, and the remaining 863 were used for the database for the support vector machine (SVM) analysis. This flowchart is described in Fig. 1.

### Statistical Analysis

Continuous clinicopathological variables were expressed as the median and range. The data were analyzed using the Mann–Whitney *U* test and Fisher exact test or Pearson’s chi-square test, depending on data characteristics.

Diagnostic accuracy was analyzed using machine learning as follows. First, partial least square (PLS) regression was performed for all 863 peak intensities to visually understand the difference in distribution between IPMN-LGD and Ad-IPMN. PLS regression is a statistical method used to project high-dimensional data into a series of linear subspaces of the explanatory variables. In this study, each serum sample had 863 explanatory variables (i.e., 863 peak intensities after data processing), and new one-dimensional variables that discriminate IPMN-LGD from Ad-IPMN are made by combining the coefficients for all 863 variables. The most discriminating new variable is defined as component 1, and the second most discriminating one is defined as component 2.^22^ Second, a discrimination test was performed using SVM. The variables used for the analysis by SVM were determined as follows. The explanatory variables (represented as *m/z*) that differed between IPMN-LGD and Ad-IPMC groups for the objective variables were identified by Student’s *t*-test and sorted by *P*-value. The SVM model was optimized by sequentially adding the corresponding explanatory variables in order from the one with the lowest *P*-value. The optimized SVM model determined the possibility score of each serum sample as a continuous value between 0 and 1, corresponding to IPMN-LGD and Ad-IPMN, respectively. The possibility score is shown as a box plot graph in Supplementary Fig. 4. To calculate the diagnostic accuracy, the threshold value used for the judgment was set to 0.5. If the value was closer to 1 than 0.5, the sample was diagnosed as IPMN-LGD, and if it was closer to 0 than 0.5, it was diagnosed as Ad-IPMN. This possibility score was evaluated using a random sub-sampling method, a type of cross validation. Finally, the receiver operating characteristic curve (ROC) according to the possibility value was described, and the area under the curve (AUC) was calculated.

Furthermore, the discriminant accuracy of the diagnostic algorithm was validated using 7 independent serum samples, which were obtained from patients who underwent pancreatectomy in 2022. In the validation set, we applied the same 130 variables obtained from training set analysis for SVM. Sensitivity, specificity, and accuracy were analyzed in the same way for seven samples.

Statistical analyses were performed using SPSS Statistics, version 25.0 (IBM Corp., Armonk, NY, USA) and EZR (The R Foundation for Statistical Computing, Vienna, Austria), and hierarchical analysis was conducted using MetaboAnalyst 5.0 (Xia Lab) [Nature protocols (2011), 6, 743-760, Jianguo Xia and David S Wishart]. *P* < 0.05 was considered statistically significant.

## Results

### Patient Backgrounds

Samples were collected from the University of Tokyo (*n* = 26) and the University of Yamanashi (*n* = 16). Seventeen patients were diagnosed with IPMN-LGD, while 25 patients were diagnosed with Ad-IPMN. Preoperative chemotherapy was not administered for any participants. In the endoscopic ultrasonography exam, an enhanced mural nodule was identified in 64.7% of IPMN-LGD, and 76.0% of Ad-IPMN. Of the entire cohort, cytology was performed in 27 patients. Atypical cells (more than class 3) were found in 70.0% of IPMN-LGD (7/10), and 94.1% of Ad-IPMN (16/17). Of the patient who underwent cytology (10 patients in IPMN-LGD and 17 patients in Ad-IPMN), three patients in LGD-IPMN and five patients in Ad-IPMN were evaluated for KRAS mutation, and two patients in LGD-IPMN and four patients in Ad-IPMN presented a mutation of KRAS status. The clinicopathological backgrounds of all patients are described in Table 1. No variables were significantly different between IPMN-LGD and Ad-IPMN patients, except for the carcinoembryonic antigen value (median IPMN-LGD 1.7 and Ad-IPMN 2.6, *P* = 0.003).

**Table 1:** Clinicopathological backgrounds

Variables	IPMN-LGD	Ad-IPMN	*p*
	*n*=17	*n*=25	
Age, years	72 (49-84)	71 (49–83)	0.729
Sex, male(%)	8 (47.1)	17 (68.0)	0.175
CEA, ng/ml	1.7 (1.1–13.4)	2.6 (1.1–114.0)	0.003
CA19-9, U/ml	9.0 (1.0–58.0)	11.5 (1.0–863.0)	0.158
Triglyceride, mg/dl	126 (52–354)	110 (44–512)	0.894
Co-morbidity, *n*,(%)			
Diabetes mellitus	4 (23.5)	11 (44.0)	0.174
Dyslipidemia	7 (41.2)	6 (24.0)	0.237
Hypertension	6 (35.3)	10 (40.0)	0.758
Synchronous other cancer	2 (11.8)	3 (12.0)	0.406
IPMN type, MD/BD/Combined	2/6/9	6/5/14	0.424
Malignant sign, HRS/WF	13/4	20/5	0.784
Enhanced mural nodule, yes(%)	11 (64.7)	19 (76.0)	0.426
Atypical cells (cytology ≥ class 3), yes (%)	7 (70.0)	16 (94.1%)	0.084
KRAS mutation, yes(%)	2 (66.6)	4 (80.0)	0.809
Surgical procedure, *n*,(%)			
Pancreaticoduodenectomy	9 (52.9)	15 (60.0)	0.650
Distal pancreatectomy	7 (41.2)	7 (28.0)	0.374
Total pancreatectomy	1 (5.9)	3 (12.0)	0.507
Ad-IPMN invasiveness, *n*,(%)			
Non-invasive	–	16 (64.0)	–
Invasive	–	9 (36.0)	–

*p* value was calculated by Mann-Whitney *U* test or chi-square test

IPMN, intraductal papillary mucinous neoplasm; LGD, low-grade dysplasia; Ad, Advancedadvanced; CEA, carcinoembryonic antigen; CA19-9, carbohydrate antigen 19-9; MD, main -duct; BD, branched -duct; HRS, high- risk stigmata; WF, worrisome feature

### Data Acquired by PESI-MS and Processing

**Fig. 2:**
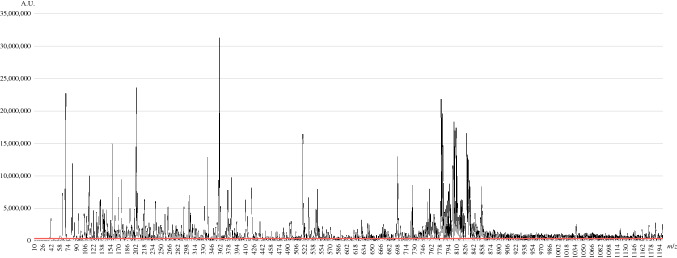
The mass spectrum composed of 863 average peak intensities was plotted. The *red line* is drawn at the 500,000 A.U. level. The *m/z* values with peak intensities lower than this *red line* were excluded from the analysis. *A.U.,* arbitrary units

Using PESI-MS, 1191 peak intensities from each serum sample were acquired. Among them, 328 peak intensities were excluded because these average intensities were less than 500,000 A.U. The remaining 863 peak intensities were integrated for further analysis. The mass spectrum comprising all average peak intensities is presented in Fig. 2. The red line in this figure shows the 500,000 A.U. level. The *m/z* values with peak intensities lower than this red line were excluded from the analysis.

### PLS Regression Analysis

**Fig. 3:**
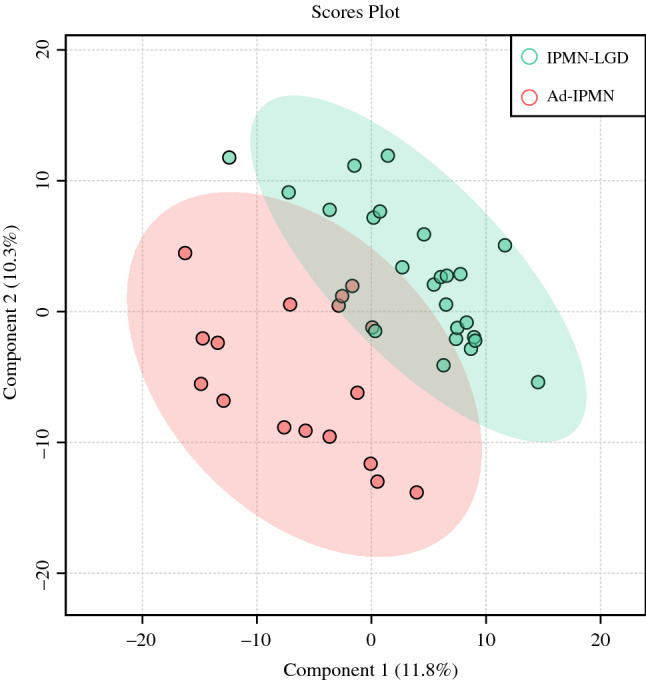
Partial least squares regression. The *pink plot* reflects IPMN-LGD, and the *green plot* indicates Ad-IPMN. *Component 1* and *component 2* were weighted 12.1% and 9.8% of all components. *Pink* and *green* areas were the 95% confidence intervals of each plot. *IPMN*, intraductal papillary mucinous neoplasm; *LGD*, low-grade dysplasia; *Ad*, Advanced

A two-dimension plot generated by PLS regression is shown in Fig. 3. Although the plotted regions of IPMN-LGD and Ad-IPMN partially overlapped, they were well separated by component 1 and 2 macroscopically.

### SVM and Cross Validation

**Fig. 4:**
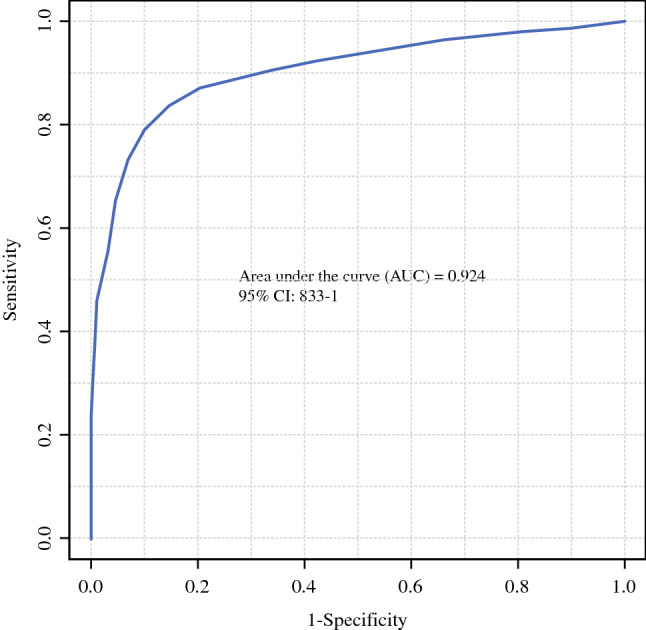
Receiver operating characteristic curve of probabilities by discriminant analysis. By discriminating the spectral data using a support vector machine with the top 130 variables identified by Student’s *t*-test, the AUC was 0.924. *CI*, confidence interval; *AUC*, area under the curve

The ROC curve generated by SVM is presented in Fig. 4. The selected number of variables for SVM was 130. The *m/z* of these 130 variables and the corresponding *P*-values of the Student’s *t*-test are shown in Supplementary Table 1. The AUC generated by the SVM model using the database of the 130 variables was 0.924 (95% confidence interval: 0.831–0.981). The specificity, sensitivity, and diagnostic accuracy calculated according to the possibility scores of cross validations using the repeat random sub-sampling method were 88.2% (15/17), 88.0% (22/25), and 88.1% (37/42), respectively. We show these scores for each group with box plot graphs in Supplementary Fig. 4A.

### Validation Analysis

In the validation set, one patient was classified into IPMN-LGD, and 6 patients were classified into Ad-IPMN by the pathological diagnosis. In total, 5 out of 7 serum samples were correctly diagnosed by our method, the sensitivity was 66.7% (4/6), the specificity was 100.0% (1/1), and the accuracy rate was 71.4% (5/7). The box plot of possibility score is shown in Supplementary Fig. 4B. The possibility score of IPMN-LGD was quite low, 0.00003.

## Discussion

This study demonstrated that the combination of PESI-MS and machine learning had the potential to detect IPMN canceration by analyzing a small amount of patient serum. The two-dimensional figure provided by PLS regression enabled the visualization of the two diseases separately. When 130 parameters out of the entire dataset acquired by PESI-MS were applied to the database for SVM, the system showed that the discrimination rate of Ad-IPMN from IPMN-LGD was 88.1%, and the AUC was 0.924. In the validation set, sensitivity, specificity, and accuracy were 66.7, 100.0, and 71.4%, respectively. Additionally, this diagnostic tool required ~10 min without special preparation other than drawing patient blood. Conversely, HRS and WF proposed in the International Consensus Guideline in 2018 require invasive examination and have a diagnostic accuracy of 63–75%. This diagnostic tool has a high ability to diagnose IPMN canceration, providing a promising diagnostic modality in clinical practice.

Although histopathology remains the gold standard for diagnosing cancer, integrated molecular analysis using mass spectrometry has gained attention in recent years for detecting malignant tumors because of its high throughput ability, sensitivity and specificity.^23,24^ Unlike other mass spectrometry methods, PESI-MS provides a spectrum by ionizing molecules with a distinct probe motion, which requires minimal sample preparation, thereby addressing the time-consuming processing of mass spectrometry.^15^ Other recent methods of mass spectrometry, including matrix assisted laser desorption/ionization-mass spectrometry, desorption electrospray ionization-mass spectrometry or MasSpec Pen, have produced precise results and promising biomarkers; however, they require complex pretreatment and are not suitable for routine medical care.^25,26^ In this study, PESI-MS analysis, data processing and machine learning required ~10 min in total, which is sufficient for clinical situations, such as outpatient examination or rapid intraoperative diagnosis. Preoperative pathological diagnosis of IPMN canceration is difficult oncologically. PESI-MS analysis is considered meaningful from that point of view.

This study is also significant in that only serum was used for the sample rather than malignant tissue. The collection of tumor tissue is not minimally invasive. Additionally, the concept of intratumoral heterogeneity has recently become widely accepted.^27^ Thus, examinations using tumor tissue, including needle biopsy, can lead to false-negative results. In contrast, serum samples can be obtained with no invasiveness, and their components do not change regardless of where the serum sample is collected. Accordingly, this diagnostic modality is easy to repeat and may have high reproducibility.

The calculation method with maximization of separating margins is named SVM, a type of machine learning. The classification feature of SVM is expanding its use mainly in cancer genomics.^28^ Multi-omics data obtained from clinical specimens contain a lot of information, and it is possible to extract latent features that lead to various clinical questions and the elucidation of pathological conditions by performing integrated analysis in combination with medical information. However, the omics data obtained from clinical samples have large individual differences and variations in distribution between samples, and it is difficult to ensure the robustness and semantic interpretability of results. Therefore, it requires optimal variable selection to maximize differences between two targeted factors. In this study, PESI-MS analysis first provided 11,910 ion intensity values, which were reduced to one value for one integer, resulting in 1191 peak intensities by data processing as described above. Among them, variables with the largest difference were selected in the comparative test, and the number of variables that maximized the difference was determined. Finally, 130 variables were selected. Previous studies selected SVM variables using the same approach and demonstrated a good discriminative ability with reproducibility.^29,30^ An additional important point to note regarding the interpretation is that the greater the number of variables, the higher the probability of overfitting. In this study, cross validation was performed to decrease overfitting.^31^ Further, analysis of the validation set gave an accuracy rate of 71.4%. Although it was lower than the training set analysis, perhaps due to small samples, a probability score of 0.00003 for IPMN-LGD was a notable value. Therefore, this diagnostic modality may provide a good specificity for IPMN-LGD, which could avoid over-surgery for benign IPMN patients.

In this study, the largest difference between IPMN-LGD and Ad-IPMN was identified at an *m/z* of 1035 (see Supplementary Table 1). According to the Human Metabolome Database version 5.0, this *m/z* is composed of phosphatidylinositol phosphates, asparagoside F, uttronin A, and triglycerides.^32^ Considering that phosphatidylinositol phosphates act intracellularly, and asparagoside F and uttronin A are present in plants, triglycerides were a representative molecule at this *m/z*. High serum triglyceride concentrations have been considered to be associated with metabolic syndrome. A few cohort studies revealed that they were related to a risk of colon, breast, or cervical cancer.^33-35^ As shown in Fig. 2, although the peak intensity of *m/z* 1035 was not large among all plots, a slight difference in these molecules was detected by PESI-MS analysis. The ROC curve drawn by only *m/z* 1035 is shown in Supplementary Fig. 3. The AUC was 0.775, which was equivalent to the value reported by previous clinical research. ^11-13^ However, by adding 129 peak intensities that were almost statistically different between IPMN-LGD and Ad-IPMN, the AUC increased to 0.924, considerably higher than that seen in previous reports. It should be noted that *m/z* 1035 did not reflect actual serum triglycerides (ref. Table 1). For example, both *m/z* 1035 and *m/z* 1024 (13^th^ lower *P*-value in Supplementary Table 1) consist of triglycerides. That is, PESI-MS discriminates finer part differences in triglycerides.

There are several limitations to this work. First, this study included a small sample of patients. In general, small size prediction research should be accepted only to show the potential of new biological insights. Although this research applied cross validation to reduce overfitting, more data and validation by other datasets are needed for future clinical application. Second, the Ad-IPMN group in this study included both carcinoma in situ and invasive adenocarcinoma. Invasive adenocarcinoma can change serum components more significantly. Third, when multiple comparisons are performed, the false discovery rate should be controlled. Supplementary Table 1 also shows a “q value” calculated according to the Benjamini-Hochberg procedure. If the significant criterion is set as 0.05, all q values were more than significant criteria. Thus, this study cannot conclude the difference between IPMN-LGD and Ad-IPMN as for the molecular identification.

## Conclusions

The new diagnostic system consisting of PESI-MS and SVM discriminated Ad-IPMN from IPMN-LGD with a high accuracy using the top 130 variables with a potential biomarker. Variable selection using more data will increase the robustness of this diagnostic tool and facilitate its clinical application.

## Supplementary Information

Below is the link to the electronic supplementary material.

Supplementary file1 (DOCX 1036 KB)

Supplementary file2 (DOCX 19 KB)

## Data Availability

The data that support the findings of this study are available on reasonable request from the corresponding author.
