# Supplementary figures and images for: New Diagnostic Modality Combining Mass Spectrometry and Machine Learning for the Discrimination of Malignant Intraductal Papillary Mucinous Neoplasms
Source: Ann Surg Oncol. 2023 Jan 8;30(5):3150–7. doi: 10.1245/s10434-022-13012-y (PMC10085898; doi:10.1245/s10434-022-13012-y)

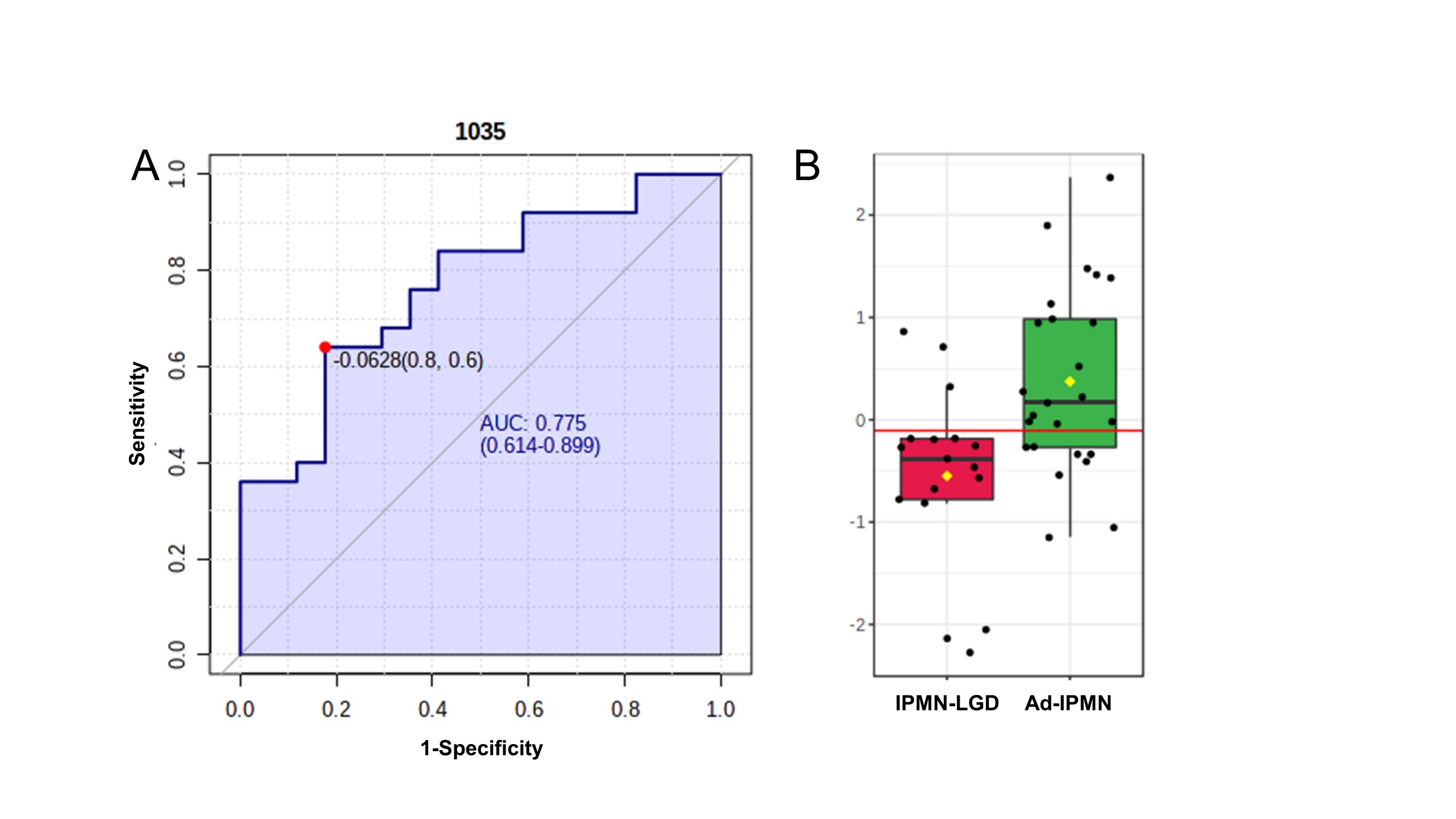

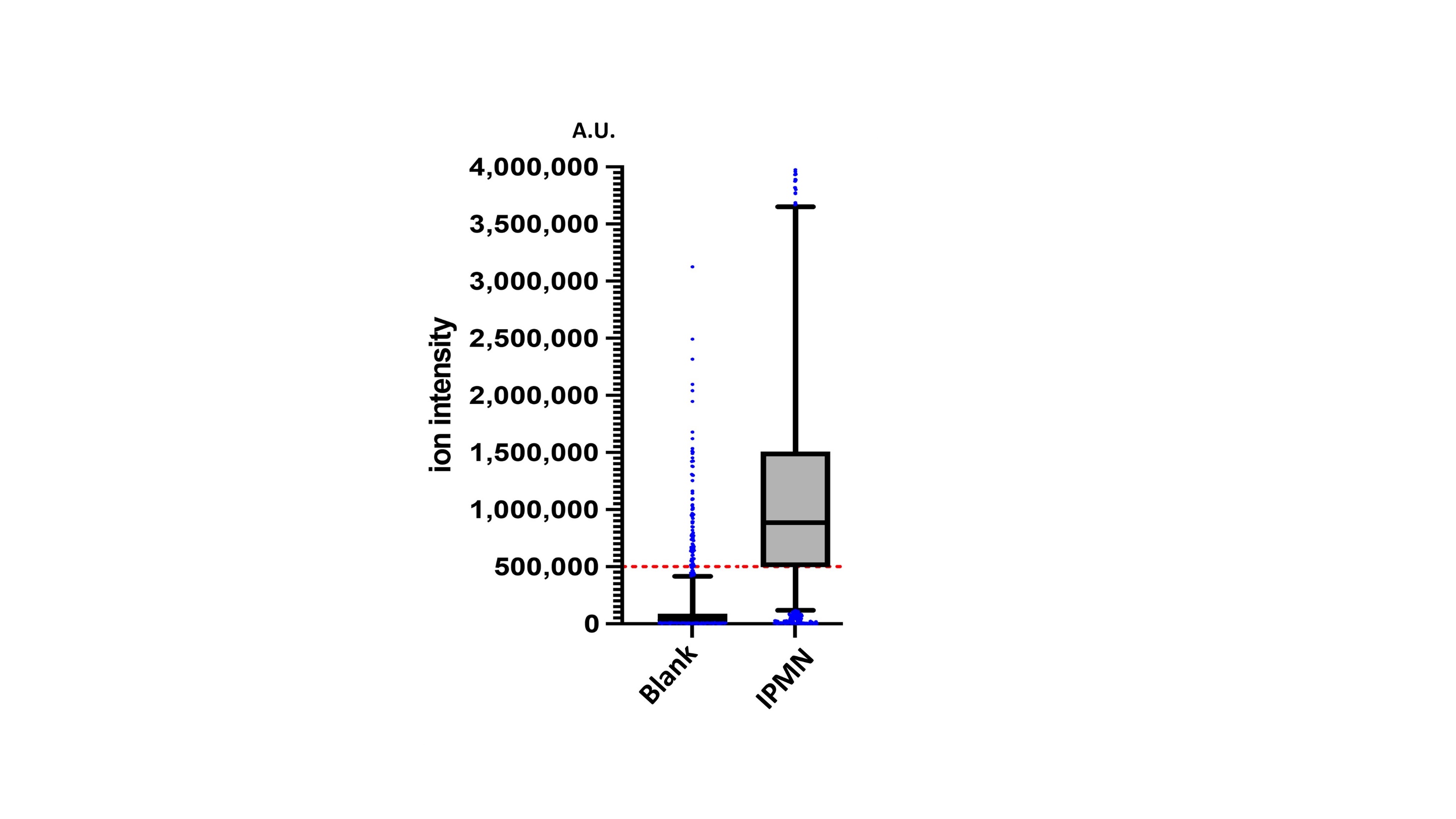

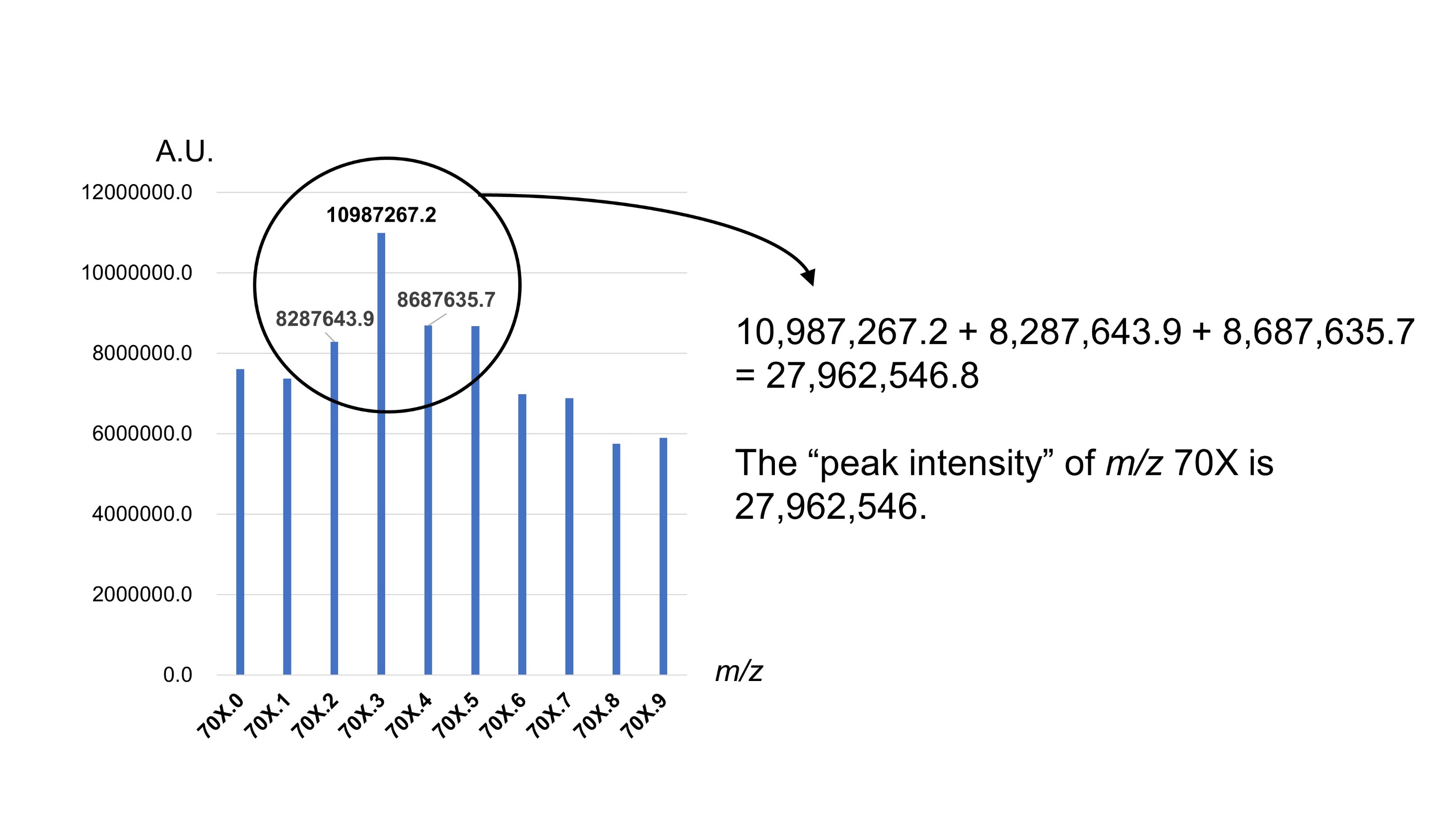

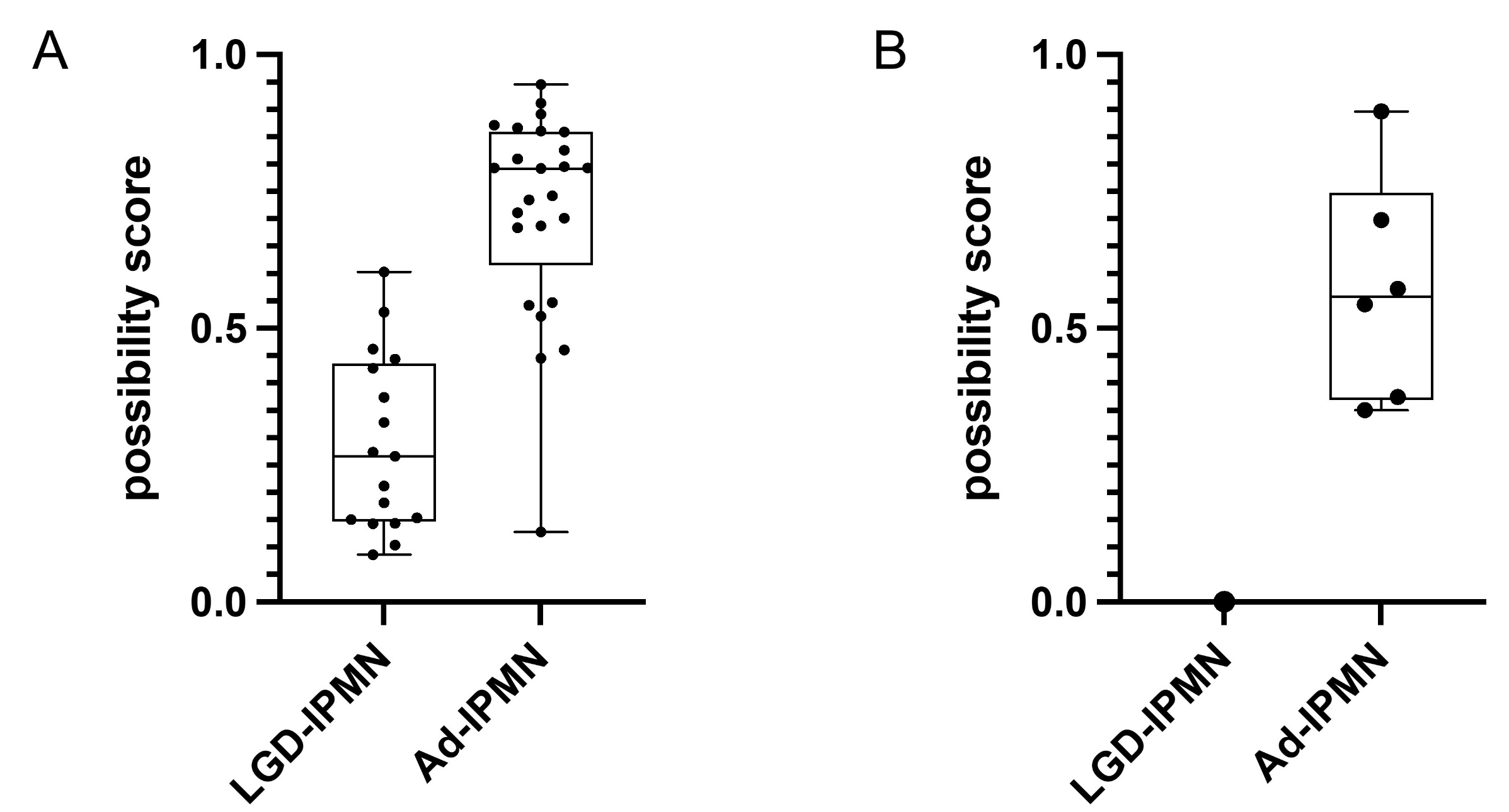

Supplement: Supplementary file 1 — Supplementary file1 (DOCX 1036 KB) [file 10434_2022_13012_MOESM1_ESM.docx]
