# Supplementary material for: New Diagnostic Modality Combining Mass Spectrometry and Machine Learning for the Discrimination of Malignant Intraductal Papillary Mucinous Neoplasms
Source: Ann Surg Oncol. 2023 Jan 8;30(5):3150–7. doi: 10.1245/s10434-022-13012-y (PMC10085898; doi:10.1245/s10434-022-13012-y)
Supplement: Supplementary file 2 — Supplementary file2 (DOCX 19 KB) [file 10434_2022_13012_MOESM2_ESM.docx]

**Supplementary Table 1. Two sample t test for each *m/z*.**

| *m/z* | p value | q value | FC (=log_2_(IPMN-LGD peak ion intensity/Ad-IPMN peak ion intensity)) |
| --- | --- | --- | --- |
| 1035 | 0.003061 | 0.3978988 | -0.78801 |
| 616 | 0.004236 | 0.2753615 | -1.1337 |
| 998 | 0.004862 | 0.2106858 | 0.11792 |
| 452 | 0.006826 | 0.2218327 | 0.31593 |
| 817 | 0.006847 | 0.1780251 | -0.8616 |
| 314 | 0.007916 | 0.1715094 | 1.0267 |
| 1133 | 0.013961 | 0.2592817 | -0.97437 |
| 1089 | 0.014564 | 0.2366592 | 0.24444 |
| 136 | 0.016802 | 0.2426906 | 1.346 |
| 69 | 0.019437 | 0.2526761 | -0.091139 |
| 978 | 0.019649 | 0.2322124 | -0.70989 |
| 178 | 0.020004 | 0.2167153 | 0.0025109 |
| 1024 | 0.021329 | 0.2132887 | 0.22964 |
| 147 | 0.023128 | 0.2147555 | 0.026709 |
| 953 | 0.02337 | 0.2025414 | 0.23443 |
| 933 | 0.024134 | 0.1960909 | 0.22082 |
| 243 | 0.024945 | 0.1907542 | -0.10114 |
| 1044 | 0.025965 | 0.1875268 | -0.84711 |
| 101 | 0.026285 | 0.1798422 | 0.012569 |
| 211 | 0.027185 | 0.1767009 | -0.06183 |
| 819 | 0.028975 | 0.179369 | -0.58452 |
| 470 | 0.029401 | 0.1737318 | -0.038889 |
| 80 | 0.029733 | 0.1680538 | -0.019802 |
| 816 | 0.029918 | 0.162058 | -0.48281 |
| 1078 | 0.032147 | 0.1671633 | 0.29573 |
| 872 | 0.032842 | 0.1642085 | -0.4069 |
| 745 | 0.03441 | 0.165677 | -0.5374 |
| 179 | 0.036325 | 0.1686527 | -0.077883 |
| 93 | 0.037374 | 0.1675371 | 0.084314 |
| 1060 | 0.037942 | 0.1644139 | 0.13111 |
| 780 | 0.038264 | 0.16046 | -0.18553 |
| 210 | 0.03868 | 0.1571368 | 0.13789 |
| 751 | 0.038793 | 0.1528225 | -0.49187 |
| 132 | 0.038931 | 0.1488524 | 0.026431 |
| 867 | 0.04028 | 0.1496102 | -0.35458 |
| 195 | 0.040377 | 0.145807 | -0.026372 |
| 148 | 0.041592 | 0.1461351 | 0.34383 |
| 781 | 0.042034 | 0.1438007 | -0.16739 |
| 988 | 0.042414 | 0.1413802 | -0.86276 |
| 124 | 0.042522 | 0.1381952 | -0.030341 |
| 63 | 0.043514 | 0.1379706 | 0.040311 |
| 192 | 0.044222 | 0.1368773 | 0.38087 |
| 109 | 0.044311 | 0.1339627 | 0.15125 |
| 1062 | 0.044549 | 0.1316215 | 0.069595 |
| 686 | 0.044891 | 0.1296854 | 0.1129 |
| 908 | 0.045194 | 0.1277225 | 0.028422 |
| 315 | 0.045751 | 0.1265446 | -0.84959 |
| 275 | 0.045804 | 0.1240531 | -0.097682 |
| 162 | 0.046997 | 0.1246872 | -0.030028 |
| 844 | 0.047578 | 0.1237034 | -0.44901 |
| 836 | 0.047651 | 0.1214629 | -0.54381 |
| 727 | 0.048961 | 0.1224021 | -0.49131 |
| 1136 | 0.049116 | 0.120473 | 0.33866 |
| 362 | 0.049932 | 0.1202071 | -0.49184 |
| 642 | 0.049973 | 0.1181185 | -0.90731 |
| 141 | 0.050157 | 0.116437 | 0.031714 |
| 804 | 0.05046 | 0.1150849 | -0.16635 |
| 163 | 0.050468 | 0.1131186 | -0.085267 |
| 716 | 0.051088 | 0.1125677 | -0.86299 |
| 1180 | 0.051785 | 0.1122019 | -0.97384 |
| 1130 | 0.053733 | 0.1145129 | 0.34668 |
| 188 | 0.054107 | 0.1134504 | -0.056496 |
| 647 | 0.055277 | 0.1140633 | 0.1377 |
| 592 | 0.057689 | 0.1171814 | -0.003607 |
| 873 | 0.057918 | 0.1158357 | -0.4839 |
| 1108 | 0.058041 | 0.1143227 | -0.90242 |
| 1113 | 0.0595 | 0.1154484 | 0.20328 |
| 769 | 0.061258 | 0.1171101 | -0.4349 |
| 1080 | 0.061934 | 0.1166872 | -0.74247 |
| 1121 | 0.062102 | 0.1153321 | -0.95008 |
| 806 | 0.062808 | 0.1150009 | -0.17742 |
| 191 | 0.062833 | 0.113448 | -0.093308 |
| 1061 | 0.063428 | 0.1129535 | -0.86765 |
| 1016 | 0.063519 | 0.1115878 | -0.56911 |
| 177 | 0.064359 | 0.1115552 | -0.054387 |
| 992 | 0.064874 | 0.1109684 | -0.66056 |
| 999 | 0.065508 | 0.1105982 | -0.63694 |
| 203 | 0.066648 | 0.1110802 | -0.18971 |
| 1083 | 0.067645 | 0.1113153 | -0.54516 |
| 1135 | 0.068004 | 0.1105058 | 0.22375 |
| 169 | 0.068356 | 0.1097071 | -0.098051 |
| 669 | 0.069099 | 0.1095466 | -0.48208 |
| 376 | 0.069126 | 0.1082698 | -0.4541 |
| 422 | 0.069482 | 0.1075312 | -0.63628 |
| 430 | 0.069826 | 0.1067933 | -0.047237 |
| 977 | 0.070376 | 0.1063819 | 0.15324 |
| 214 | 0.07044 | 0.1052556 | 0.0086211 |
| 167 | 0.070657 | 0.1043792 | -0.065317 |
| 976 | 0.070867 | 0.1035129 | -0.4608 |
| 837 | 0.071225 | 0.102881 | -0.77565 |
| 86 | 0.071451 | 0.1020725 | -0.12356 |
| 1036 | 0.074214 | 0.1048676 | -0.63406 |
| 182 | 0.074223 | 0.1037526 | 0.1369 |
| 245 | 0.074623 | 0.1032014 | -0.14897 |
| 240 | 0.075122 | 0.1027986 | 0.27293 |
| 808 | 0.076028 | 0.1029544 | -0.18258 |
| 1186 | 0.076643 | 0.1027179 | -1.0932 |
| 791 | 0.076782 | 0.101854 | -1.072 |
| 171 | 0.078375 | 0.102917 | -0.10176 |
| 474 | 0.079208 | 0.1029702 | 0.068949 |
| 982 | 0.079679 | 0.1025575 | -0.64359 |
| 187 | 0.079821 | 0.1017326 | -0.05209 |
| 154 | 0.081559 | 0.102938 | -0.059326 |
| 1000 | 0.083213 | 0.1040159 | -0.046342 |
| 870 | 0.084033 | 0.1040412 | -0.36399 |
| 267 | 0.08603 | 0.1055086 | -0.11565 |
| 39 | 0.086754 | 0.1054025 | -0.13395 |
| 814 | 0.087759 | 0.1056358 | -0.46014 |
| 966 | 0.088175 | 0.1051632 | -0.076067 |
| 782 | 0.08877 | 0.1049103 | -0.20012 |
| 868 | 0.089518 | 0.1048414 | -0.33036 |
| 155 | 0.089667 | 0.104078 | 0.01938 |
| 229 | 0.090475 | 0.1040861 | -0.018422 |
| 821 | 0.091283 | 0.1040942 | -0.43654 |
| 161 | 0.091607 | 0.1035562 | -0.11006 |
| 787 | 0.092175 | 0.1032997 | 0.48499 |
| 822 | 0.092474 | 0.1027492 | -0.43854 |
| 906 | 0.095521 | 0.1052349 | -0.047446 |
| 165 | 0.098183 | 0.1072591 | 0.40711 |
| 183 | 0.098856 | 0.1070945 | -0.094075 |
| 606 | 0.100509 | 0.107985 | -0.54501 |
| 1022 | 0.102154 | 0.1088527 | -0.09016 |
| 818 | 0.102397 | 0.1082242 | -0.41247 |
| 1099 | 0.102696 | 0.1076649 | 0.12377 |
| 805 | 0.104198 | 0.1083661 | -0.17665 |
| 643 | 0.105032 | 0.1083659 | -0.49532 |
| 768 | 0.105484 | 0.1079761 | -0.46976 |
| 365 | 0.106405 | 0.108068 | 0.58595 |

FC, fold change; IPMN, intraductal papillary mucinous neoplasm; LGD, low-grade dysplasia; Ad, Advanced.

*”q value” was calculated according to the Benjamini-Hochberg procedure.
